# The Establishment of China Bronchiectasis Registry and Research Collaboration (BE-China): Protocol of a prospective multicenter observational study

**DOI:** 10.1186/s12931-022-02254-9

**Published:** 2022-12-03

**Authors:** Yong-Hua Gao, Hai-Wen Lu, Bei Mao, Wei-Jie Guan, Yuan-Lin Song, Yuan-Yuan Li, Dao-Xin Wang, Bin Wang, Hong-Yan Gu, Wen Li, Hong Luo, Ling-Wei Wang, Fan Li, Feng-Xia Guo, Min Zhang, Zhi-Jun Jie, Jing-Qing Hang, Chao Yang, Tao Ren, Zhi Yuan, Qing-Wei Meng, Qin Jia, Yu Chen, Rong-Chang Chen, Jie-Ming Qu, Jin-Fu Xu

**Affiliations:** 1 Department of Respiratory and Critical Care Medicine, Shanghai Pulmonary Hospital, Institute of Respiratory Medicine, Tongji University School of Medicine, 507 Zhengmin Road, Shanghai, 200433 China; 2 State Key Laboratory of Respiratory Disease, National Clinical Research Center for Respiratory Disease, Guangzhou Institute of Respiratory Health, The First Affiliated Hospital of Guangzhou Medical University, Guangzhou, China; 3 Department of Pulmonary and Critical Care Medicine, Zhongshan Hospital, Fudan University, Shanghai, China; 4 Department of Pulmonary and Critical Care Medicine, Xiangya Hospital, Central South University, Changsha, Hunan China; 5 Department of Pulmonary and Critical Care Medicine, The Second Affiliated Hospital of Chongqing Medical University, Chongqing, China; 6 Department of Pulmonary and Critical Care Medicine, Huzhou Central Hospital, Huzhou, Zhejiang China; 7 Department of Pulmonary and Critical Care Medicine, The Sixth People’s Hospital of Nantong, Nantong, Jiangsu, China; 8 Key Laboratory of Respiratory Disease of Zhejiang Province, Department of Respiratory and Critical Care Medicine, Second Affiliated Hospital of Zhejiang University School of Medicine, Hangzhou, Zhejiang China; 9 Department of Respiratory and Critical Care Medicine, The Second Xiangya Hospital, Central South University, Changsha, Hunan China; 10 Pulmonary and Critical Care Department, Shenzhen People’s Hospital, Shenzhen Institute of Respiratory Diseases, Shenzhen, 518020 Guangdong China; 11 Department of Respiratory and Critical Care Medicine, Songjiang District Central Hospital, Shanghai, China; 12 Department of Respiratory and Critical Care Medicine, The Eighth People’s Hospital of Shanghai, Shanghai, China; 13 Department of Respiratory and Critical Care Medicine, Shanghai General Hospital, Shanghai Jiao Tong University School of Medicine, Shanghai, China; 14 Department of Respiratory and Critical Care Medicine, Shanghai Fifth People’s Hospital, Fudan University, Shanghai, China; 15 Department of Respiratory and Critical Care Medicine, Shanghai Putuo District People’s Hospital, Shanghai, China; 16 Department of Respiratory and Critical Care Medicine, Suzhou Science and Technology Town Hospital, Suzhou, China; 17 Department of Respiratory and Critical Care Medicine, Shanghai Jiao Tong University Affiliated Sixth People’s Hospital, Shanghai, China; 18 Department of Respiratory and Critical Care Medicine, Fenghua District People’s Hospital, Ningbo, Zhejiang China; 19 Department of Respiratory and Critical Care Medicine, Shangrao People’s Hospital, Shangrao, Jiangxi China; 20 Department of Respiratory and Critical Care Medicine, Shidong Hospital of Yangpu District, Shanghai, China; 21 Department of Respiratory and Critical Care Medicine, Shengjing Hospital, China Medical University, Shenyang, China; 22 Department of Respiratory and Critical Care Medicine, Ruijin Hospital, Shanghai JiaoTong University School of Medicine, Shanghai, 200025 China

**Keywords:** Bronchiectasis, Registry, Real-world study, Protocol, BE-China

## Abstract

**Background:**

Bronchiectasis is a highly heterogeneous chronic airway disease with marked geographic and ethnic variations. Most influential cohort studies to date have been performed in Europe and USA, which serve as the examples for developing a cohort study in China where there is a high burden of bronchiectasis. The Establishment of China Bronchiectasis Registry and Research Collaboration (BE-China) is designed to: (1) describe the clinical characteristics and natural history of bronchiectasis in China and identify the differences of bronchiectasis between the western countries and China; (2) identify the risk factors associated with disease progression in Chinese population; (3) elucidate the phenotype and endotype of bronchiectasis by integrating the genome, microbiome, proteome, and transcriptome with detailed clinical data; (4) facilitate large randomized controlled trials in China.

**Methods:**

The BE-China is an ongoing prospective, longitudinal, multi-center, observational cohort study aiming to recruit a minimum of 10,000 patients, which was initiated in January 2020 in China. Comprehensive data, including medical history, aetiological testing, lung function, microbiological profiles, radiological scores, comorbidities, mental status, and quality of life (QoL), will be collected at baseline. Patients will be followed up annually for up to 10 years to record longitudinal data on outcomes, treatment patterns and QoL. Biospecimens, if possible, will be collected and stored at − 80 °C for further research. Up to October 2021, the BE-China has enrolled 3758 patients, and collected 666 blood samples and 196 sputum samples from 91 medical centers. The study protocol has been approved by the Shanghai Pulmonary Hospital ethics committee, and all collaborating centers have received approvals from their local ethics committee. All patients will be required to provide written informed consent to their participation.

**Conclusions:**

Findings of the BE-China will be crucial to reveal the clinical characteristics and natural history of bronchiectasis and facilitate evidence-based clinical practice in China.

*Trial registration* Registration Number in ClinicalTrials.gov: NCT03643653

## Introduction

Non-cystic fibrosis bronchiectasis (hereafter referred to as bronchiectasis) has historically been a neglected respiratory diseases, and our current practice in the management of bronchiectasis is largely extrapolated from chronic obstructive pulmonary disease (COPD) and cystic fibrosis rather than supported by high-quality evidence [1]. However, recent epidemiological studies have clearly shown that the prevalence and incidence of bronchiectasis are quickly rising both in high and low-income countries possibly at least in part-owing to the aging populations and the extended use of high-resolution computed tomography (HRCT) [2–4]. Thus, bronchiectasis is posing an increasing burden on healthcare systems around the world with an effect on patient’s quality of life (QoL) and survival [2–5], suggesting an urgent need for better resourced research into this condition.

An upsurged interest in recent years has transformed the field of bronchiectasis. We now have clearer definitions and classifications of the disease [6], specific QoL tools [7, 8], multidimensional severity assessment tools [5, 9–11], and more robust evidence-based treatments such as airway clearance, macrolides, and inhaled antibiotics [12–17]. Everyone within the field would agree that large-scale registries with regular and long-term follow-up, such as European Multicenter Bronchiectasis Audit and Research Collaboration (EMBARC) and US bronchiectasis registry, have made a major contribution to these advances [5, 6, 12, 13, 16–19], and have clarified with greater power and accuracy the patients’ demographics, the microbiological profiles, the most common aetiologies and the burden of disease including symptoms, comorbidities, QoL and frequency of exacerbations [20–26]. However, most epidemiological data in the field of adult bronchiectasis [5, 6, 12, 13, 16–26] to date are limited to cohorts from Europe and the USA, with few data from Asian countries, including from China—the most populous country [20, 27, 28]. Recently, national registries have been established in Korea, Australia and India, which will add more evidence to the Asian phenotypes of bronchiectasis [20, 29–31]. Emerging data have shown that there may be substantial differences between Asian patients and western patients regarding the aetiology, microbiological profiles, disease severity and comorbidities [20, 29–31]. However, the characteristics of bronchiectasis may vary according to the country and socioeconomic status of the targeted population [32]. The Establishment of China Bronchiectasis Registry and Research Collaboration (BE-China) would allow us to better understand the characterization of Chinese bronchiectasis patients and test whether the phenotypes identified in western cohorts could be validated in China, which could provide valuable insights into geographical and ethnic differences of bronchiectasis.

The heterogeneity of bronchiectasis remains the greatest clinical challenge [1]. Currently, there are no licensed therapies for bronchiectasis and the successful clinical trials in bronchiectasis are still insufficient to support evidence-based interventions possibly due to the poor understanding of the pathophysiology of the disease [12–14]. Specimens are invaluable clinical research resources for genetic and molecular studies to reveal the endotype and pathogenesis. Therefore, the development of biologic resources embedded in registry as a backbone to build a repository of blood, sputum and other biological materials for use in translational research, will help us to better understand the pathophysiology of bronchiectasis, to better phenotype patients and individualize their management. EMBARC has made a substantial contribution to the translational research in bronchiectasis in the past few years [33–35], but we still have a long way to fully understand this disease or offer the evidence-based personalized management for patients with bronchiectasis. Additional state-of-art biobank, outside of western populations, is vital for further microbiome, proteome, genome and transcriptome research due to the potentially varying genetic background and molecular characteristics among patients from different geographic regions.

The BE-China is formally initiated in January 2020, which is the only large prospective, multicenter, longitudinal cohort study in adult bronchiectasis across China. The BE-China will recruit eligible individual bronchiectasis patients, and comprehensively collect their clinical data, as well as specimens with longitudinal follow-up for future research. Herein, we describe the rational and design of the BE-China registry.

## Methods and analyses

### Study design and objectives

**Fig. 1:**
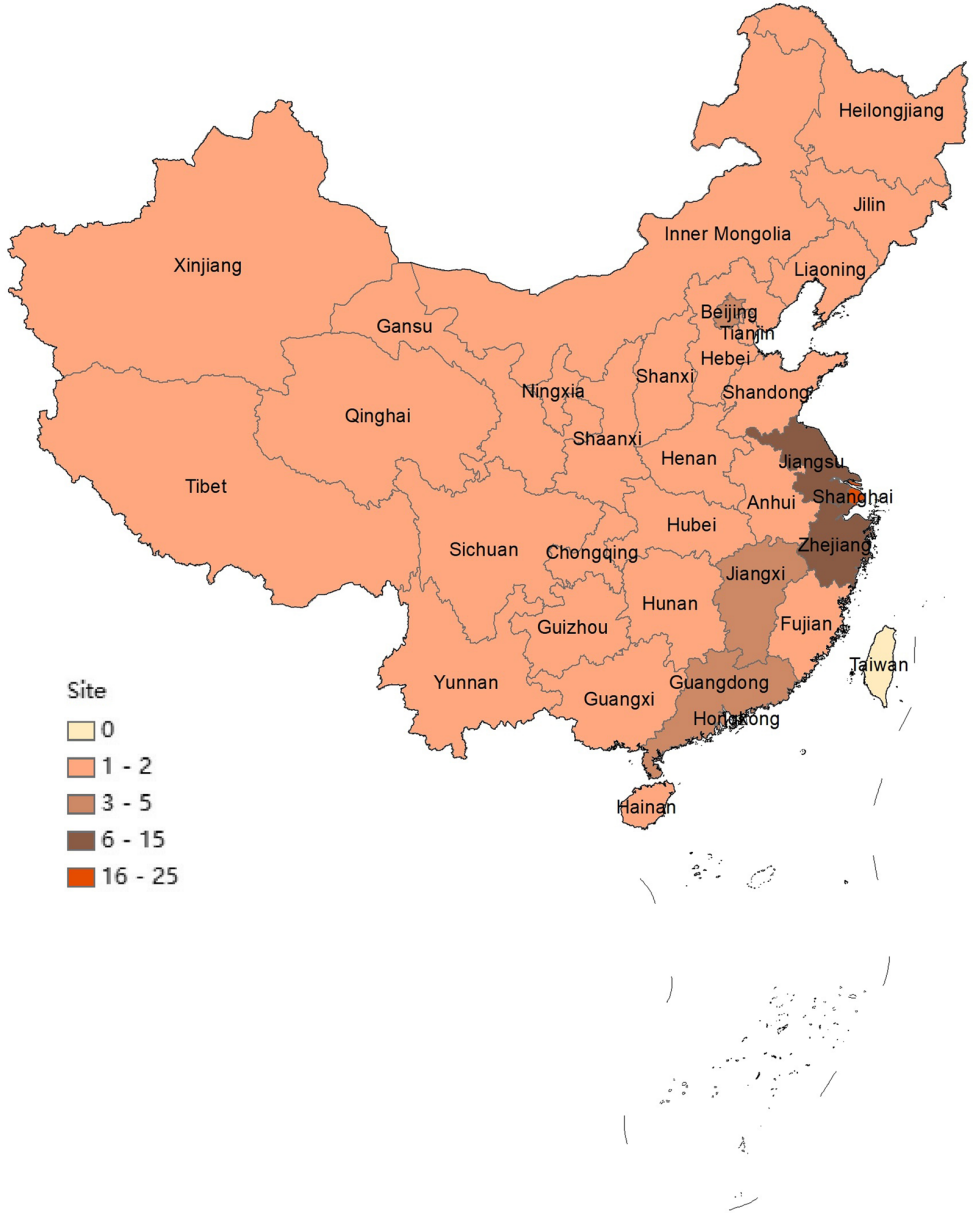
The number of 91 participating medical centers in the BE-China and their locations across China. Shanghai has enrolled twenty-five medical centers; Zhejiang enrolled fifteen medical centers; Jiangsu enrolled eleven medical centers; Guangdong enrolled five medical centers; Beijing enrolled four medical centers; Jiangxi enrolled four medical centers; Hunan enrolled two medical centers; Guangxi enrolled two medical centers; Hubei, Fujian, Guizhou, Henan, Liaoning, Ningxia, Sichuan, Xinjiang, Yunnan, Chongqing, Hebei, Shandong, Tianjin, Shaanxi, Inner Mongolia, Anhui, Gansu, Tibet, Qinghai, Heilongjiang, Jilin, Hainan, Shanxi enrolled one medical center each; Hong Kong, Macau and Taiwan did not enroll any medical center, respectively

The BE-China registry, initiated in January 2020, is a Yangtze River Delta region-based nationwide, multicenter, prospective, longitudinal, observational cohort study platform which aims to enroll a minimum of 10,000 consecutive adult patients with radiologically confirmed bronchiectasis in China. Enrolled patients are managed by trained and certified local physicians at each participating center according to clinical practice and guidelines without imposed interventions. The number of participating centers and their locations are shown in Fig. 1.

**Fig. 2:**
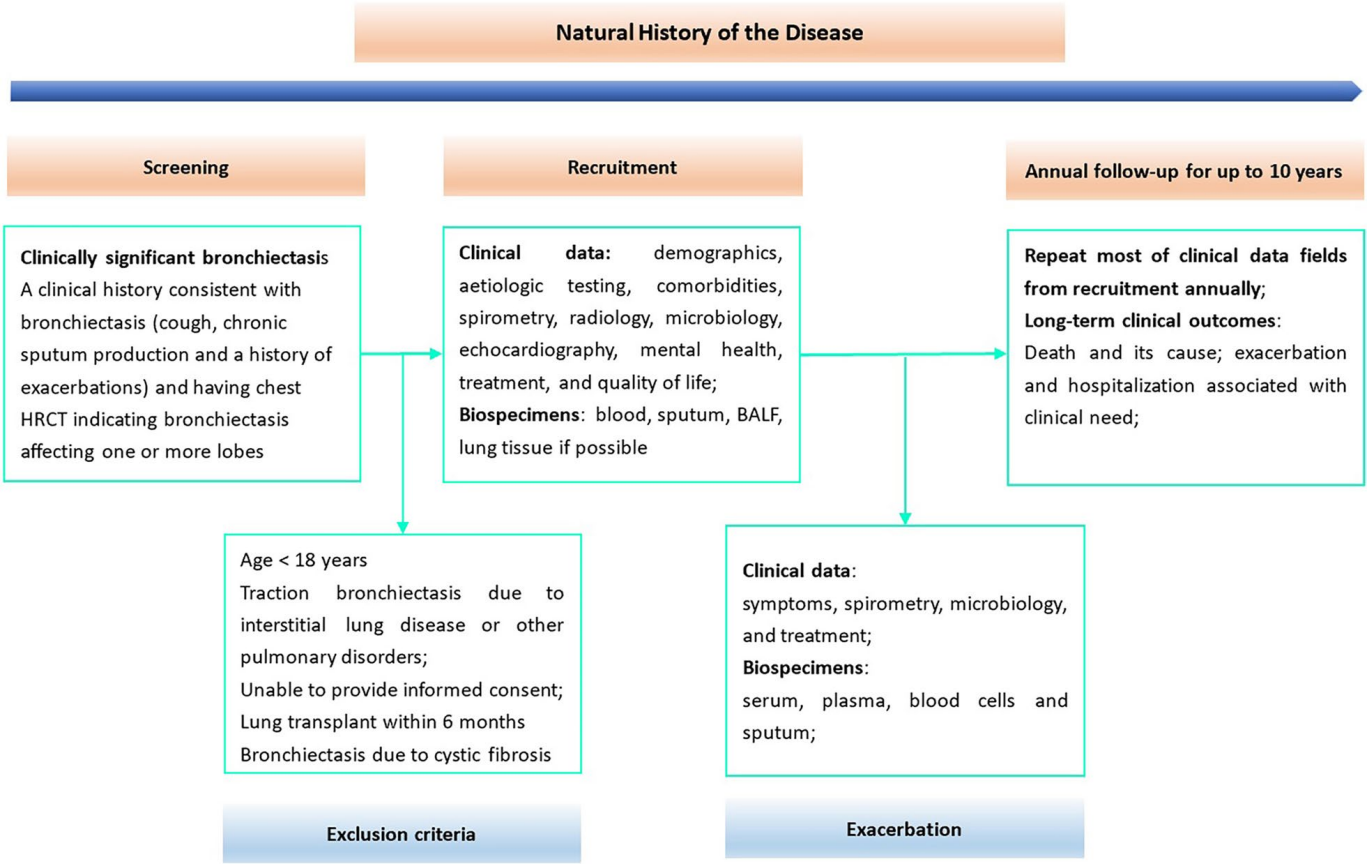
Study flowchart of the BE-China

Comprehensive data, including demographics, medical history, comorbidities, aetiological testing, lung function, echocardiography, microbiological profiles in sputum or bronchoalveolar lavage fluid (BALF), radiological scores, QoL and treatment, are collected at baseline (recruitment) during the steady-state. The patients will be followed up to 10 years on annual basis (within a 3-month variance) aligned to routine clinical attendance. In addition, the recruiting centers with access to appropriate facilities are encouraged to biobank the sputum, serum, plasma, blood cells, BALF and lung tissue, if possible, both at steady-state and exacerbation. The designs of the registry are presented in Fig. 2.

The main objectives of the BE-China are to:

1. Characterize the demographics, aetiology, lung function, microbiological profiles, exacerbation, disease severity, QoL and treatment of the Chinese bronchiectasis patients;
2. Validate the phenotype of bronchiectasis identified in the Western cohorts and figure out the differences between China and the Western countries;
3. Elucidate the risk factors associated with deteriorating QoL, rapid decline of lung function, frequent exacerbation, and mortality;
4. Follow the natural history of disease in individual aetiologies;
5. Explore the new bronchiectasis phenotype and endotype of stable disease and exacerbation through integrating the clinical data, microbiome, proteomics, and genomics in China;
6. Facilitate large-scale randomized controlled trials in China.

To address the potential ethnic and geographic differences, we have established some main predefined hypotheses. To this end, we aim to compare clinical characteristics of the population of patients who suffer from bronchiectasis between China and other regions across the world, and to perform exploratory analyses about the risk factors which may contribute to clinically important outcome measures in bronchiectasis (such as exacerbation, QoL and mortality).

### Participants, inclusion and exclusion criteria

The inclusion criteria of this registry are: (1) patients aged 18 years or older with a clinical history consistent with bronchiectasis (chronic cough, daily sputum production, and a history of exacerbations) and having performed chest HRCT in the past year indicating bronchiectasis which affects one or more lobes; (2) remaining clinically stable upon recruitment. Patients with exacerbations are allowed to be enrolled into the registry at least 4 weeks after antibiotic discontinuation.

The exclusion criteria are as follows: (1) traction bronchiectasis associated with interstitial lung disease or other pulmonary disorders; (2) cystic fibrosis associated bronchiectasis; (3) patients who are unable or unwilling to provide informed consent.

### Specimens and biobank settings

Samples will be biobanked at each individual center if appropriate facilities are available. Blood, sputum, BALF and lung tissue samples of the enrolled patients who are able to provide consent, are collected via local well-trained investigators according to critical standard operating procedure. The blood will be centrifuged, and divided into three aliquots (plasma, serum, or blood cells, respectively). Unprocessed spontaneous sputum will be aliquoted. Per protocol, among patients with diffuse bronchiectasis, BALF samples will be conducted in the right middle lobe or left lingula lobe. In the case of localized bronchiectasis, the targeted segment is chosen based on chest HRCT scan. BALF samples are centrifuged for 20 min at 3184 g at 4 °C. The supernatant is then divided into aliquots. The aliquoted blood, sputum, lung tissue, BALF supernatant and cell pellets will be stored at − 80 °C for further genetic, proteomic and microbiome analyses. Participants can still be enrolled into the registry platform even if they are unable to provide samples.

### Data collection

Three parts of data are collected in BE-China Registry: (1) baseline data; (2) annual follow-up data; (3) data at exacerbation. The detailed information is described as follows (Table 1):

**Table 1:** Data collection at baseline, exacerbation and follow-up in the BE-China

Baseline	Annual follow-up	Exacerbations
Mandatory data collection		
Enrollment date	Follow-up data	Symptoms
Age	Age	Spirometry
Sex		Microbiological profiles
Body mass index	Body mass index	Treatment
Socioeconomic status (including education, occupation, medical insurance and annual household income)	Socioeconomic status	Blood and sputum samples if possible
Smoking history	Smoking history	
mMRC score	mMRC score	
Sputum color assessment	Sputum color assessment	
The number of exacerbation and hospitalization in the past 1 year before recruitment	The number of exacerbations and hospitalizations per patient per year	
Comorbidities	Comorbidities	
Spirometry	Spirometry	
Imaging tests	Imaging tests	
Aetiological tests	Updated medical history and laboratory tests to determine the aetiology of bronchiectasis	
Blood cell counts		
Total lgG, lgA and lgM		
Tests for ABPA (total lgE, specific lgE to Aspergillus etc.)		
Microbiological profiles	Microbiological profiles	
Chest physiotherapy	Chest physiotherapy	
Respiratory medications	Respiratory medications	
Specific aetiological treatment	Specific aetiological treatment	
Vaccination status	Vaccination status	
Gastroesophageal reflux disease questionnaire		
Quality of life-Bronchiectasis Questionnaire	Quality of life-Bronchiectasis Questionnaire	
Bronchiectasis Health Questionnaire	Bronchiectasis Health Questionnaire	
Leicester Cough Questionnaire	Leicester Cough Questionnaire	
Hospital Anxiety and Depression Scale	Hospital Anxiety and Depression Scale	
Recommended data collection		
Autoantibody tests (ANA, ENA and ANCA)		
Complement C3 and C4		
α1-antitrypsin deficiency: serum α1-antitrypsin and genetic tests		
Cystic fibrosis: sweating test and genetic tests		
Ciliary function test: nasal FeNO, high-speed video analysis, transmission electron microscopy, genetic testing		
FeNO		
6 min walking distance		
Arterial blood gas		
Echocardiography		
Serum, plasma, blood cells, sputum, lung tissue		

*ABPA* allergic bronchopulmonary aspergillosis, *ANA* anti-nuclear antibodies, *ANCA* antineutrophil cytoplasmic antibodies, *ENA* antibodies to extractable nuclear antigen, *lg* immunoglobin, *FeNO* fractional exhaled nitric oxide, *mMRC* modified British medical research council

1. **Demographic data** age, sex, body-mass index (BMI), ethnic groups (such as Han, Hui, Tibetan, Mongol, Uyghur and so on), the highest educational level, occupational status, medical insurance and annual household income. The highest educational level is stratified into the following groups: illiteracy, elementary school graduate, junior high school graduate, senior high school graduate or equivalent, and college graduate or above. The occupational categories include manual workers, farmers, unemployed individuals, businessmen, clerks, professionals, managers, government employees or others where occupations should be clearly recorded. The annual household income is recorded in the total RenMinBi (RMB). The medical insurance of each patient is recorded in the following groups: no medical insurance, new rural cooperative medical insurance, commercial medical insurance, urban resident medical insurance, urban employee medical insurance.
2. **Health-related QoL**
  The registry will use both “QoL-Bronchiectasis” questionnaire (QoL-B) and Bronchiectasis Health Questionnaire (BHQ) to evaluate QoL [7, 8, 36]. Both questionnaires are the disease-specific QoL tools that have been specially developed and validated for use in bronchiectasis. The Hospital Anxiety and Depression Scale is employed to evaluate the status of anxiety and depression in patients with bronchiectasis [37]. Gastroesophageal reflux disease questionnaire (Gerd-Q) is used to diagnose the gastroesophageal reflux disease in patients with bronchiectasis as the potential aetiology or comorbidity [38]. An automatic calculator tool of these questionnaires is incorporated into the registry platform to aid in the calculation.
3. **Leicester Cough Questionnaire (LCQ)**
  We have included the LCQ in our registry because it has been validated for use in bronchiectasis [39, 40]. Cough has been rated as one of the most troublesome symptom in bronchiectasis by most patients in a survey of 711 European patients with bronchiectasis [41]. Inclusion of this questionnaire will also allow comparison to other datasets.
4. **Aetiology of bronchiectasis**
  The physicians caring for patients will determine the aetiology of bronchiectasis based on medical history, extensive aetiological testing, and questionnaires. Items of aetiological testing will follow the recommendation by national and international guidelines [12–14], with blood cell counts, serum immunoglobulins (lg) (total lgG, lgA and lgM), and testing for allergic bronchopulmonary aspergillosis (ABPA) (total serum lgE, specific lgE to Aspergillus etc.) as the mandatory testing. Other aetiologial tests, including autoimmune disease, alpha one antitrypsin deficiency, cystic fibrosis, primary ciliary dyskinesia, are carried out in patients with suggestive clinical features determined by local physicians.
5. **Exacerbations**
  We define the exacerbations based on the criteria recommended by EMBARC consensus [42], in which exacerbations are defined as a deterioration of three or more following symptoms for at least 48 h which require an immediate change of routine treatment: (1) cough; (2) sputum volume increase and/or consistent change; (3) sputum purulence; (4) dyspnea and/or exercise intolerance; (5) fatigue and/or malaise; (6) hemoptysis. Severe exacerbations are defined as exacerbations requiring an emergency room visit or hospitalization.
6. **Sputum assessment**
  Sputum colour is assessed using a validated photographic sputum colour chart that is graded as 1 (mucoid) to 4 (highly purulent) [43]. Sputum volume (mL/day) is estimated by patients’ report.
7. **Spirometry**
  Pre-bronchodilator and post-bronchodilator spirometry are performed according to American Thoracic Society (ATS)/European Respiratory Society (ERS) guidelines [44]. The percentage of predicted values for forced expiratory volume in one second (FEV_1_) and forced vital capacity (FVC) will be calculated by using a reference equation of Chinese people. In addition, total lung capacity, residual volume, inspiratory capacity, small airway function, or diffusion function assessed by spirometry are also collected when available.
8. **Microbiological profiles**
  The microbiological profiles from any sample (sputum, BALF or induced sputum) will be recorded either at steady-state or exacerbation. In addition, the data of fungi and non-tuberculous mycobacteria, including individual species, will be also collected.
9. **Disease severity**
  Both the Bronchiectasis Severity Index (BSI) and E-FACED are employed to assess the severity of bronchiectasis based on each variables [5, 9–11], with the total scores being calculated automatically at database platform.
10. **Comorbidities**
  Both pulmonary and extrapulmonary comorbidities are carefully collected [23]. Regarding pulmonary comorbidities, we record the presence or absence of physician-diagnosed asthma, COPD, rhinitis, chronic sinusitis and nasal polyp. Extrapulmonary comorbidities, including cardiovascular diseases, stroke, digestive diseases, endocrine diseases, hematological diseases, renal diseases, rheumatoid diseases, malignancy (including tumor sites) and immunodeficiency types will be recorded.
11. **Radiology**
  We use both the modified Reiff score and Bhalla score to assess the radiological severity of bronchiectasis [5, 45]. Physicians in each center determine the radiological score independently after careful training. Furthermore, images of chest CT scans at specific sites are required to be uploaded to the database platform for future use.
12. **Echocardiography**
  Echocardiography, which is recommended but not mandated, will be used to evaluate the left and right cardiac function in bronchiectasis. Patients with cardiac dysfunction or pulmonary hypertension are at greater risk of death in bronchiectasis [46, 47], which are commonly seen in China.
13. **Treatments**
  Regular treatment associated with bronchiectasis are recorded in detail, including airway clearance techniques, antibiotics (oral, inhaled, or nebulized), mucoactive drugs (oral or nebulized), long-acting muscarinic antagonist (LAMA), long-acting β2 agonist (LABA), inhaled corticosteroids (ICS), ICS/LABA, LABA/LAMA, ICS/LABA/LAMA. Data on the use of long-term home oxygen therapy, non-invasive ventilation, intravenous immunoglobulin, ABPA related treatment (oral corticosteroids, anti-fungi drugs), along with the vaccination status are also collected.

### Follow-up

Participants enrolled in the registry will be prospectively followed up on an annual basis (± 3 months) which is aligned to the routine clinical attendance to collect longitudinal data, including the changes in medication use, QoL, the number of exacerbation and hospitalization, lung function and survival data (including both death and lung transplant). In addition, patients are encouraged to contact their physicians when they experience an exacerbation during follow-up. Clinical data (including symptoms, lung function, microbiology in sputum and the use of medication) and specimens (including blood and sputum samples) before antibiotic prescription at exacerbation, are collected if possible (Fig. 2).

### Quality control, data management and monitoring

The registry collects data via an electronic data capture (EDC) solution which is designed based on the electronic case report form (eCRF). Patient information is de-identified and confidentially collected and then entered into the BE-China platform which was built on a secure website (www.chinabronchiectasis.com). All electronic data are protected by account and passwords.

Several strategies are undertaken to manage the data and ensure quality control. First, the EDC system and eCRF are designed with the built-in logic checks during data entry. Second, each eligible center should designate one or two knowledgeable clinical research coordinators, who have been trained by centralized coordinators according to research protocols, to collect and record patient data in the EDC system to ensure the completeness and accuracy of each entered data. Third, data quality is monitored by a dedicated project and data management team according to predefined procedures. Once abnormal or missing values are detected, queries will be sent by the EDC system automatically to local investigators to check and revise the data. Moreover, research and data quality control report will be generated and circulated to all participating centers and local investigators periodically by Email or Wechart software. Each local investigators can access their own data without restrictions. However, complete data analysis requires submission of a research proposal to the BE-China Scientific Committee, and then access can be granted after approval of the research proposal.

### Ethics and dissemination

Patients will receive regular follow-up at the outpatient clinic or via telephone by the local investigators. All patients will be required to sign written informed consent by the ethics committee according to the Declaration of Helsinki and local regulatory polices at each center. The BE-China has been registered in the clinical trials registry (www.clinicaltrials.gov) with an identifier of NCT03643653. The BE-China study group will follow the recommendations regarding authorship provided by the International Committee of Medical Journal Editors. The findings will be disseminated via publication in peer-reviewed journals, conference presentations, or academic website.

## Discussion

This is a nationwide prospective multicenter ongoing registry in adults with bronchiectasis, which is formally launched in January 2020 in mainland China. Until November 2021, 3758 patients have been recruited. The main objectives of the registry aim to describe the disease spectrum and natural history of bronchiectasis by long-term follow-up, as well as to reveal the phenotypes/endotypes and facilitate large randomized controlled trials among the Chinese bronchiectasis patients.

To date, seven ongoing large-scale registries have been established in Europe as well as in individual countries in the past decade (Table 2) [18–21, 48, 49], which have significantly improved our understanding of bronchiectasis. EMBARC, which was established in 2012 [18], represents the first and largest truly international bronchiectasis network around the world, and thus far it has enrolled more than 19,000 patients who are planned to be followed-up for 5 years. Since its establishment, EMBARC investigators have published more than 70 papers spanning from aetiology, endophenotypes to disease management of bronchiectasis [19, 22–26, 33–35, 50], and the first international guideline for the management of bronchiectasis [12]. The success of EMBRAC highlights the importance of establishing the large-scale bronchiectasis registry with long-term follow up to uncover the heterogeneity of the disease, which could not be achieved by single-center small sample studies. The registries in India and Korea have demonstrated substantial geographic and ethic difference of the disease between Asian and Western countries [20, 29–31]. However, the registries in Asia, do not collect biologic specimens and only enroll a limited number of participants. In addition, previous clinical trials in bronchiectasis, such as RESPIRE [51, 52], have demonstrated that patients from different geographic region behaved differently to what was expected with devastating consequences for the trial. This illustrates that the more data about geographic and ethnic differences of bronchiectasis should be clearly addressed.

**Table 2:** Summary of six ongoing large registry studies on adults with bronchiectasis around the world

Registry	Main objectives	Settings	Study population	Sample size	Centralized biobank	Enrollment timeline	Follow-up
EMBARC	To develop a pan-European, multicentre bronchiectasis registry incorporating baseline data collection with annual follow-up data for at least 5 years; to describe the demographics, comorbidities, aetiology, medication usage, resource consumption, exacerbations, microbiology, severity and prognosis of bronchiectasis across Europe; to facilitate multinational cooperation, within and outwith Europe; to facilitate the creation of national registries in European countries that currently do not have a bronchiectasis research infrastructure	A minimum of 20 European countries	Adults with a clinical history consistent with bronchiectasis and computed tomography demonstrating bronchiectasis affecting one or more lobes	Estimated to enroll 10,000 patients by March 2020, and has enrolled more than 19,000 patients as of October 2021	Yes	2012–ongoing	Up to 5 years
KMBARC	To describe of the clinical characteristics, including patient demographics, phenotype, aetiology, progression, treatment and prognosis, of Korean patients with bronchiectasis; to evaluate of disease burden, including use of medication and medical resources, acute exacerbation, hospitalisation and mortality, in Korean patients with bronchiectasis; to evaluate of the rare aetiology of bronchiectasis (e.g., allergic bronchopulmonary aspergillosis, rheumatoid arthritis and tuberculosis); to elucidation of risk factors associated with acute exacerbation and prognosis	More than 26 hospitals in South Korea	Adults with computed tomography demonstrating bronchiectasis affecting one or more lobes regardless the presence of respiratory symptoms or not	At least 1200 patients over the study period	No	August 2018–ongoing	Up to 5 years
BronchUK	To develop a multicentre bronchiectasis registry incorporating baseline data collection with annual follow-up data for at least 5 years; to facilitate the creation of a biobank in bronchiectasis to underpin future mechanistic studies; to describe the treatment patterns across the UK, phenotypic data, comorbidities and healthcare use; to facilitate multinational cooperation, especially with EMBARC, within academia and with industry to develop new discoveries; to develop key partnerships with experts not currently working in bronchiectasis to optimally use the datasets	At least nine secondary care centres within UK	Adults with a clinical history consistent with bronchiectasis and computed tomography demonstrating bronchiectasis	A minimum of 1500 patients	Yes	November 2014–ongoing	A maximum of 5 years
US Registry	To support collaborative research and assist in the planning of multi-center clinical trials for the treatment of NTM and non-CF Bronchiectasis; to provide better insight into the study of the different types of Bronchiectasis, as well as the pathophysiology of the disorder	17 active sites in the United States	Adults with a physician-established diagnosis of bronchiectasis	Estimated to enroll 5000 patients, and has enrolled more than 4000 patients as of October 2021	Unknown	2007–ongoing	Up to 20 years
Australian Registry	To understand the cause, incidence and prevalence of bronchiectasis; to explore the burden of illness and of treatment; to support the exploration of innovative treatments; to improve quality of life and offer opportunities for consumer engagement; to identify the economic impact of bronchiectasis on an individual and our community; to maximise equity of access for all Australians to bench marked, evidence-based management for bronchiectasis	At least 14 sites across the Australian mainland	Australian adults and children with a physician diagnosis of bronchiectasis with abnormal bronchial dilatation demonstrated on computed tomography chest scan	Unknow	Unknown	2015–ongoing	Up to 5 years
Indian Registry	To advance research and improve clinical care for patients with non-cystic fibrosis bronchiectasis in India	At least 31 centres across India	Adults with a clinical history consistent with bronchiectasis and computed tomography demonstrating bronchiectasis affecting one or more lobes	Unknow (has enrolled more than 2195 patients as of September 2017)	Unknown	May 2015–ongoing	Up to 5 years
RIBRON	To obtain information from bronchiectasis patients to improve the knowledge of the disease in Spain; to facilitate and promote multicenter and multidisciplinary research in bronchiectasis; to identify groups of patients who may be candidates for future clinical trials; to assess the follow-up of Spanish bronchiectasis clinical guide-lines and recommendations in the attempt to standardize and improve patient management	43 hospitals located throughout Spain	Adults with a clinical picture consistent with bronchiectasis of any etiology (including cystic fibrosis) diagnosed with chest high-resolution computed tomography	Unknow (has enrolled more than 2300 patients as of July 2019)	No	February 2015–ongoing	Unknow

The designated functions of the BE-China could compensate above unaddressed gaps. This registry comprehensively collects the clinical data of all enrolled patients from steady-state to exacerbation, as well as during annual follow-up, which makes the descriptions of the natural history of the disease feasible and practical. Disease characteristics and progression patterns will be compared among different subgroup patients stratified by demographics (e.g., age, gender, BMI) or clinical features (e.g., aetiology, disease severity, comorbidities). In addition, risk factors that contribute to poor outcomes (deteriorated QoL, progressive lung function decline, frequent exacerbation, and mortality) and the corresponding predicted tool will be developed and validated. Furthermore, we will compare the clinical characteristics, progression patterns and management of bronchiectasis between China and Western countries, or other Asian countries by international collaborations. Also, we will test whether the phenotype and endotype identified in western populations can be validated in Chinese patients. The substantial heterogeneity of bronchiectasis underscores the importance of a better mechanistic understanding of its pathology and progression. The biobanking of samples matched with the detailed clinical data, will allow us to conduct mechanistic research in the future. In short, we believe the BE-China will not only promote bronchiectasis research in China, but also facilitate multidisciplinary collaborative research around the world.

Certain limitations should be acknowledged. Since the patients will be mainly enrolled from secondary and tertiary hospitals, it appears that patients may not represent the full profiles of bronchiectasis across China. However, the referral system in China is not strict, and patients can go directly to public hospitals for all outpatients care. The representation of patients will be not a major concern. In addition, similar with any other registry study, withdrawal of patients and missing data may result in bias and there may be other unidentified or unmeasured confounding factors.

## Conclusions

In conclusion, BE-China will establish a rich set of clinical and biological database on a large cohort of well-characterized individuals with bronchiectasis in China. The registry will provide unique and detailed insight into disease characteristics and progression patterns among Chinese patients and lay the foundation for international collaboration in the future.

## Data Availability

Not applicable.

## Abbreviations

ABPA: Allergic bronchopulmonary aspergillosis
ATS: American Thoracic Society
BALF: Bronchoalveolar lavage fluid
BE-China: The Establishment of China Bronchiectasis Registry and Research Collaboration
BHQ: Bronchiectasis Health Questionnaire
BSI: Bronchiectasis Severity Index
COPD: Chronic obstructive pulmonary disease
eCRF: Electronic case report form
EDC: Electronic data capture
EMBARC: European Multicenter Bronchiectasis Audit and Research Collaboration
ERS: European Respiratory Society
FEV_1_: Forced expiratory volume in one second
FVC: Forced vital capacity
Gerd-Q: Gastroesophageal reflux disease questionnaire
HRCT: High-resolution computed tomography
ICS: Inhaled corticosteroids
lg: Immunoglobulin
IQR: Interquartile range
LAMA: Long-acting muscarinic antagonist
LABA: Long-acting β2 agonist
LCQ: Leicester Cough Questionnaire
QoL: Quality of life
SD: Standard deviation

## References

[1] Chalmers JD, Chang AB, Chotirmall SH (2018). Bronchiectasis. Nat Rev Dis Prim.

[2] Quint JK, Millett ERC, Joshi M (2016). Changes in the incidence, prevalence and mortality of bronchiectasis in the UK from 2004 to 2013: a population-based cohort study. Eur Respir J.

[3] Ringshausen FC, Roux AD, Diel R (2015). Bronchiectasis in Germany: a population-based estimation of disease prevalence. Eur Respir J.

[4] Feng J, Sun L, Sun X (2022). Increasing prevalence and burden of bronchiectasis in urban Chinese adults, 2013–2017: a nationwide population-based cohort study. Respir Res.

[5] Chalmers JD, Goeminne P, Aliberti S (2014). The bronchiectasis severity index. An international derivation and validation study. Am J Respir Crit Care Med.

[6] Aliberti S, Goeminne PC, O'Donnell AE (2022). Criteria and definitions for the radiological and clinical diagnosis of bronchiectasis in adults for use in clinical trials: international consensus recommendations. Lancet Respir Med.

[7] Quittner AL, Marciel KK, Salathe MA (2014). A preliminary qualify of life questionnaire-bronchiectasis: a patient-reported outcome measure for bronchiectasis. Chest.

[8] Spinou A, Siegert RJ, Guan WJ (2017). The development and validation of the bronchiectasis health questionnaire. Eur Respir J.

[9] Martínez-García MÁ, de Gracia J, VendrellRelat M (2014). Multidimensional approach to non-cystic fibrosis bronchiectasis: the FACED score. Eur Respir J.

[10] Martinez-Garcia MA, Athanazio RA, Girón R (2017). Predicting high risk of exacerbations in bronchiectasis: the E-FACED score. Int J Chron Obstruct Pulmon Dis.

[11] Wang H, Ji XB, Li CW (2018). Clinical characteristics and validation of bronchiectasis severity score systems for post-tuberculosis bronchiectasis. Clin Respir J.

[12] Polverino E, Goeminne PC, McDonnell MJ (2017). European respiratory society guidelines for the management of adult bronchiectasis. Eur Respir J.

[13] Hill AT, Sullivan AL, Chalmers JD (2019). British thoracic society guideline for bronchiectasis in adults. Thorax.

[14] Bronchiectasis Expert Consensus Writing Group, Pulmonary Infection Assembly, Chinese Thoracic Society (2021). Expert consensus on the diagnosis and treatment of adult bronchiectasis in China. Zhonghua Jie He He Hu Xi Za Zhi.

[15] Muñoz G, de Gracia J, Buxó M (2018). Long-term benefits of airway clearance in bronchiectasis: a randomized placebo-controlled trial. Eur Respir J.

[16] Chalmers JD, Boersma W, Lonergan M (2019). Long-term macrolide antibiotics for the treatment of bronchiectasis in adults: an individual participant data meta-analysis. Lancet Respir Med.

[17] Laska IF, Crichton ML, Shoemark A (2019). The efficacy and safety of inhaled antibiotics for the treatment of bronchiectasis in adults: a systematic review and meta-analysis. Lancet Respir Med.

[18] Chalmers JD, Aliberti S, Polverino E (2016). The EMBARC European bronchiectasis registry: protocol for an international observational study. ERJ Open Res.

[19] Aliberti S, Polverino E, Chalmers JD (2018). The European multicentre bronchiectasis audit and research collaboration (EMBARC) ERS clinical research collaboration. Eur Respir J.

[20] Dhar R, Singh S, Talwar D (2019). Bronchiectasis in India: results from the European multicentre bronchiectasis audit and research collaboration (EMBARC) and respiratory research network India registry. Lancet Glob Health.

[21] Aksamit TR, O'Donnell AE, Barker A (2017). Adult patients with bronchiectasis: a first look at the US bronchiectasis research registry. Chest.

[22] Lonni S, Chalmers JD, Goeminne PC (2015). Etiology of non-cystic fibrosis bronchiectasis in adults and its correlation to disease severity. Ann Am Thorac Soc.

[23] Araújo D, Shteinberg M, Aliberti S (2018). The independent contribution of *Pseudomonas aeruginosa* infection to long-term clinical outcomes in bronchiectasis. Eur Respir J.

[24] McDonnell MJ, Aliberti S, Goeminne PC (2016). Comorbidities and the risk of mortality in patients with bronchiectasis: an international multicentre cohort study. Lancet Respir Med.

[25] Gao YH, Abo Leyah H, Finch S (2020). Relationship between symptoms, exacerbations, and treatment response in bronchiectasis. Am J Respir Crit Care Med.

[26] Chalmers JD, Aliberti S, Filonenko A (2018). Characterization of the “frequent exacerbator phenotype” in bronchiectasis. Am J Respir Crit Care Med.

[27] Wang N, Qu JM, Xu JF (2018). Bronchiectasis management in China, what we can learn from European respiratory society guidelines. Chin Med J.

[28] Lin JL, Xu JF, Qu JM (2016). Bronchiectasis in China. Ann Am Thorac Soc.

[29] Lee H, Choi H, Sim YS (2020). KMBARC registry: protocol for a multicentre observational cohort study on non-cystic fibrosis bronchiectasis in Korea. BMJ Open.

[30] Lee H, Choi H, Chalmers JD (2021). Characteristics of bronchiectasis in Korea: first data from the Korean multicenter bronchiectasis audit and research collaboration registry and comparison with other international registries. Respirology.

[31] Visser SK, Bye PTP, Fox GJ (2019). Australian adults with bronchiectasis: the first report from the Australian bronchiectasis registry. Respir Med.

[32] Chandrasekaran R, Mac Aogáin M, Chalmers JD (2018). Geographic variation in the aetiology, epidemiology and microbiology of bronchiectasis. BMC Pulm Med.

[33] Finch S, Shoemark A, Dicker AJ (2019). Pregnancy zone protein is associated with airway infection, neutrophil extracellular trap formation, and disease severity in bronchiectasis. Am J Respir Crit Care Med.

[34] Keir HR, Shoemark A, Dicker AJ (2021). Neutrophil extracellular traps, disease severity, and antibiotic response in bronchiectasis: an international, observational, multicohort study. Lancet Respir Med.

[35] Dicker AJ, Lonergan M, Keir HR (2021). The sputum microbiome and clinical outcomes in patients with bronchiectasis: a prospective observational study. Lancet Respir Med.

[36] Guan WJ, Xu JF, Luo H (2022). A double-blind randomized placebo-controlled phase 3 trial of tobramycin inhalation solution in adults with bronchiectasis with Pseudomonas aeruginosa infection. Chest.

[37] Gao YH, Guan WJ, Zhu YN (2018). Anxiety and depression in adult outpatients with bronchiectasis: associations with disease severity and health-related quality of life. Clin Respir J.

[38] Guan WJ, Gao YH, Xu G (2015). Aetiology of bronchiectasis in Guangzhou, southern China. Respirology.

[39] Murray MP, Turnbull K, MacQuarrie S (2009). Validation of the Leicester cough questionnaire in non-cystic fibrosis bronchiectasis. Eur Respir J.

[40] Gao YH, Guan WJ, Xu G (2014). Validation of the Mandarin Chinese version of the Leicester cough questionnaire in bronchiectasis. Int J Tuberc Lung Dis.

[41] Aliberti S, Masefield S, Polverino E (2016). Research priorities in bronchiectasis: a consensus statement from the EMBARC clinical research collaboration. Eur Respir J.

[42] Hill AT, Haworth CS, Aliberti S (2017). Pulmonary exacerbation in adults with bronchiectasis: a consensus definition for clinical research. Eur Respir J.

[43] Murray MP, Pentland JL, Turnbull K (2009). Sputum color: a useful clinical tool in non-cystic fibrosis bronchiectasis. Eur Respir J.

[44] Miller MR, Hankinson J, Brusasco V (2005). Standardisation of spirometry. Eur Respir J.

[45] Bedi P, Chalmers JD, Goeminne PC (2018). The BRICS (bronchiectasis radiologically indexed CT score): a multicenter study score for use in idiopathic and postinfective bronchiectasis. Chest.

[46] Alzeer AH, Al-Mobeirek AF, Al-Otair HA (2008). Right and left ventricular function and pulmonary artery pressure in patients with bronchiectasis. Chest.

[47] Wang L, Jiang S, Shi J (2016). Clinical characteristics of pulmonary hypertension in bronchiectasis. Front Med.

[48] De Soyza A, Mawson P, Hill AT (2021). BronchUK: protocol for an observational cohort study and biobank in bronchiectasis. ERJ Open Res.

[49] Martinez-García MA, Villa C, Dobarganes Y (2021). RIBRON: the Spanish online bronchiectasis registry. Characterization of the first 1912 patients. Arch Bronconeumol.

[50] Chalmers JD, Haworth CS, Metersky ML (2020). Phase 2 trial of the DPP-1 inhibitor brensocatib in bronchiectasis. N Engl J Med.

[51] De Soyza A, Aksamit T, Bandel TJ (2018). RESPIRE 1: a phase III placebo-controlled randomised trial of ciprofloxacin dry powder for inhalation in non-cystic fibrosis bronchiectasis. Eur Respir J.

[52] Aksamit T, De Soyza A, Bandel TJ (2018). RESPIRE 2: a phase III placebo-controlled randomised trial of ciprofloxacin dry powder for inhalation in non-cystic fibrosis bronchiectasis. Eur Respir J.

